# Clinical outcomes of kidney transplants on patients with end-stage renal disease secondary to lupus nephritis, polycystic kidney disease and diabetic nephropathy

**Published:** 2016-03-30

**Authors:** John Fredy Nieto-Ríos, Lina María Serna-Higuita, Sheila Alexandra Builes-Rodriguez, Ricardo Cesar Restrepo-Correa, Arbey Aristizabal-Alzate, Catalina Ocampo-Kohn, Angélica Serna-Campuzano, Natalia Cardona-Díaz, Nelson Darío Giraldo-Ramirez, Gustavo Adolfo Zuluaga-Valencia

**Affiliations:** 1 Sección Nefrología. Hospital Pablo Tobon Uribe, Medellín, Colombia; 2 Sección de Nefrología, Universidad de Antioquia, Medellín, Colombia; 3 Sección de Pediatría y Puericultura, Universidad de Antioquia. Medellin, Colombia; 4 Sección de Nefrología, Universidad Pontificia Bolivariana, Medellín, Colombia; 5 Sección de Medicina Interna, Hospital Pablo Tobón Uribe. Medellín, Colombia

**Keywords:** Kidney transplantation, Lupus nephritis, diabetic nephropathies, polycystic kidney diseases

## Abstract

**Background:**

Patients with lupus nephritis could progress to end-stage renal disease (10-22%); hence, kidney transplants should be considered as the treatment of choice for these patients.

**Objective:**

To evaluate the clinical outcomes after kidney transplants in patients with chronic kidney diseases secondary to lupus nephritis, polycystic kidney disease and diabetes nephropathy at Pablo Tobon Uribe Hospital.

**Methods:**

A descriptive and retrospective study performed at one kidney transplant center between 2005 and 2013.

**Results:**

A total of 136 patients, 27 with lupus nephritis (19.9%), 31 with polycystic kidney disease (22.8%) and 78 with diabetes nephropathy (57.4%), were included in the study. The graft survivals after one, three and five years were 96.3%, 82.5% and 82.5% for lupus nephritis; 90%, 86% and 76.5% for polycystic kidney disease and 91.7%, 80.3% and 67.9% for diabetes nephropathy, respectively, with no significant differences (*p*= 0.488); the rate of lupus nephritis recurrence was 0.94%/person-year. The etiology of lupus vs diabetes vs polycystic disease was not a risk factor for a decreased time of graft survival (Hazard ratio: 1.43; 95% CI: 0.52-3.93).

**Conclusion:**

Kidney transplant patients with end stage renal disease secondary to lupus nephritis has similar graft and patient survival success rates to patients with other kidney diseases. The complication rate and risk of recurrence for lupus nephritis are low. Kidney transplants should be considered as the treatment of choice for patients with end stage renal disease secondary to lupus nephritis.

## Abstract (es)

**Antecedentes:**

Pacientes con nefritis lúpica pueden progresar a enfermedad renal crónica terminal (10-22%); en estos pacientes el trasplante renal debe ser considerado como la terapia de elección. Objetivo: Evaluar los desenlaces clínicos de un grupo de pacientes con enfermedad renal crónica terminal por nefropatía lúpica, enfermedad renal poliquística y nefropatía diabética que fueron sometidos a trasplante renal en el Hospital Pablo Tobón Uribe.

**Métodos:**

Estudio retrospectivo, descriptivo, realizado en un solo centro de trasplante renal, durante el período 2005-2013.

**Resultados:**

Se evaluaron 136 pacientes: 27 con nefritis lúpica (19.9%), 31 con enfermedad renal poliquística (22.8%) y 78 con nefropatía diabética (57.4%). La supervivencia del injerto a uno, tres y cinco años fue de de 96.3%, 82.5% y 82.5% en nefropatía lúpica, 90%, 86% y 76.5% en enfermedad renal poliquística y 91.7%, 80.3% y 67.9% en nefropatía diabética respectivamente, sin diferencias estadísticas significativas (Long Rank test= 0.488). La tasa de recurrencia de nefritis lúpica posterior al trasplante renal fue de 0.94%/persona-año. Tener lupus vs diabetes o enfermedad renal poliquística no fue un factor de riesgo para disminución del tiempo de supervivencia del injerto (Hazard ratio= 1.43; 95% IC= 0.52-3.93).

**Conclusiones:**

Los pacientes enfermedad renal crónica terminal secundaria a nefritis lúpica, que son llevados a trasplante renal tienen tasas de éxito similar en cuanto a supervivencia del injerto y del paciente, al compararlos con otras enfermedades renales. La tasa de complicaciones y el riesgo de recurrencia de la nefropatía lúpica son bajos. El trasplante renal debe ser considerado como la terapia de elección para los pacientes con enfermedad renal crónica estadio terminal secundaria a nefritis lúpica.

## Introduction

Lupus nephritis (LN) is the main cause of morbidity and mortality in patients with systemic lupus erythematous (SLE); it is present in more than half of the patients, particularly in patients of Hispanic and African Americans 1,2. Despite the advances in treatment, 10-22% of these patients progress to end-stage renal disease (ESRD) during the first 10 years of the disease 3-5. In 2010, according to a United States annual survey, 1.8% of ESRD patients in need of kidney replacement therapy (KRT) were LN patients 6,7. In a study performed in Medellin-Colombia, 77% of SLE patients had LN during the first year of the evolution of the disease 8.

Patients with LN-ESRD are generally younger than those with ESRD by other causes (35 years vs 47 years, respectively) 9; hence, kidney transplants (KTs) should be considered as the treatment of choice for these patients 1,3,4,10. Since 1959, SLE-ESRD patients have received KTs 11. Nonetheless, the incidence of graft failure and risk of recurrence of LN following a KT is very variable 12-16.

Regarding grafts and patient survival, a similar success rate in SLE-ESRD transplant patients and transplant patients from other causes have been observe 17-20; however, to date, studies are lacking in comparing them simultaneously with a group with an excellent prognosis following transplantation, such as polycystic kidney disease (PCKD) patients, and with a group with a worse prognosis, such as diabetic nephropathy (DN) patients 19-23. The main objective of this study was to evaluate the graft and patient survival in KT patients with DN-ESRD and PCKD-ESRD, conditions that have the worst and best prognosis following a KT, respectively. 

## Materials and Methods

A descriptive and retrospective study was performed in patients older than 18 years who were diagnosed with ESRD secondary to LN, DN and PCKD and who received a KT between 2005 and 2013 at Pablo Tobón Uribe Hospital (PTUH) of Medellin-Colombia. Patients younger than 18 years, those receiving simultaneous transplants and those lost during the follow-ups were excluded from the study.

The following clinical variables were evaluated in the three groups: gender, age, ethnicity, type of dialysis, time on dialysis previous to the transplant, comorbidities, type of donor, number of human leukocyte antigen (HLA) incompatibilities, cold ischemia time, induction therapy and type of immunosuppression. Patient and graft survival were the main outcomes evaluated. Other outcomes such as basal creatinine level and glomerular filtration rate (GFR) using CKD-EPI after one month, one year, three years and on the last follow-up were also reported. KT complications (such as graft dysfunction, and surgical, infectious, cardiovascular, metabolic and neoplastic complications), types of graft rejection confirmed by biopsy, and mortality associated with these causes were evaluated. It was not possible to collect the pre-transplant profile of lupus patients because during lupus relapses they were previously attended in another hospital. These clinical variables were collected from the medical records of the Kidney Transplant Service of PTUH and added to an Excel database. The Ethics Committee of PTUH approved the protocol. 

The statistical analysis was performed using SPSS^®^ version 18 software. Qualitative variables were analyzed calculating the frequencies, rates (%/person-year) and proportions. The quantitative variables were described as means or medians with their respective standard deviations or percentiles (p25-75), according to the distribution of the data identified using the Shapiro-Wilk test. ANOVA was used to compare the quantitative variables and outcome in the variables that reached normality, while the Kruskal-Wallis test was used to evaluate the non-normal variables. Chi-squared test with a level of significance of 0.05 was used for the qualitative variables. Graft and patient survival were calculated using the Kaplan-Meier method adjusted according to the etiology of ESRD (LN, DN, PCKD), and the statistical significance was verified using the log-rank test. Finally, survival was verified using Cox regression multivariate analysis, an adjusted Cox regression model was performed with the significant variables in the univariate analysis (*p* <0.2) and for the risk factors previously reported in the literature.

##  Results

Between 2005 and 2013, 642 KTs were performed at PTUH. The etiological cause of ESRD was diagnosed as NL, PCKD or ND in 144 cases: 29 as LN (4.5%), 37 as PCKD (5.8%) and 78 as DN (12.2%). Eight patients were excluded, 2 were excluded due to a simultaneous liver-KT, and 6 were lost during the follow up, and the remaining 136 patients were distributed as follows: 78 (57.4%) as DN, 31 (22.8%) as PCKD and 27 (19.9%) as SLE.

The demographic characteristics of the three groups are described in Table 1. A total of 59.6% were males; 6.9% were of African Americans; the median age at transplant was 51 years (p25-75: 40.2-57); 61.7% had a previous hemodialysis, 26.5% had peritoneal dialysis and 11.8% of the patients did not receive dialysis prior to the transplantation. The median time for dialysis was 13 months (p25-75: 7-31 months), and 6 months was recorded as the median time on the waiting list before transplantation (p25-75: 3-10 months). Additionally, 99.3% were deceased-donor transplants; 130 (95.6%) received induction therapy: 83 patients (61.5%) received alemtuzumab, 20 (14.8%) received basiliximab, 10 (7.4%) received daclizumab, and 17 (12.6%) received thymoglobulin. The cyclosporine-mycophenolate-prednisolone immunosuppression protocol was used in 47 (34.6%) patients and was the most frequently used, followed by tacrolimus-mycophenolate-prednisolone in 32 (23.5%) patients, cyclosporine-azathioprine-prednisolone in 26 (19.1%) patients and cyclosporine-prednisolone in 11 (8.1%) patients (Table 1). According to the group assessment, a higher proportion of men with DN-ESRD and PCKD-ESRD and a higher proportion of women with SLE-ESRD were observed (p<0.01). Diabetics were significantly older and had a higher incidence of coronary disease and chronic occlusive arterial disease than the NL and PCKD groups (Table 1).

**Table 1.** Demographic characteristics of the chronic kidney disease transplanted patients

Variable*	Lupus (n= 27)	Polycystic disease (n= 31)	Diabetes (n= 78)	p value**
Women	24 (88.8)	13 (41.9)	18 (23.1)	<0.01 (a)
Mean age (SD) in years	32.54 (8.52)	48.62 (8.15)	52.75 (9.45)	<0.05 (b)
Race African Americans vs No African Americans				
African Americans	2 (7.4)	1 (3.2)	7 (9)	0.58 (a)
Non-African Americans	25 (92.6)	30 (96.8)	71 (91)	
Congestive heart failure	2 (7.4)	1 (3.2)	3 (3.8)	0.69 (a)
Coronary disease	0	0	8 (10.2)	0.04 (a)
Hypertension	25 (92.6)	28 (90.3)	77 (98.7)	0.11 (a)
Cerebrovascular disease	0	1 (3.2)	2 (2.6)	0.67 (a)
Chronic occlusive arterial disease	0	0	13 (16.7)	<0.01 (a)
Median time on the waiting list (p25-75) in months	6 (3-10)	6 (4-10)	6.5 (3.25-12)	0.95 (c)
Median dialysis time prior to transplantation (p25-75) in months	20 (7.75-39.75)	12 (2-22.5)	14.5 (7.25-27.5)	0.19 (c)
Median cold ischemia time (p25-75) in hours	12 (9.75-16)	15 (11-20)	15.75 (13-20.75)	0.04 (c)
Incompatibility HLA-DR				
DR incompatibility equal to 0	4 (14.8)	2 (6.4)	5 (6.4)	0.10 (a)
DR incompatibility equal to 1	10 (37)	16 (51.6)	49 (62.8)	
DR incompatibility equal to 2	13 (50)	13 (48.1)	24 (30.8)	
Use of induction therapy	27 (100)	29 (93.5)	74 (94.9)	0.44 (a)
Type of therapy induction used				
Use of alemtuzumab	23 (85.2)	21 (67.74)	39 (50)	0.12 (a)
Use of basiliximab	2 (7.4)	3 (9.6)	15 (19.2)	
Use of daclizumab	0	2 (6.4)	8 (10.2)	
Use of thymoglobulin	2 (7.4)	3 (9.67)	12 (15.4)	
No induction	0	2 (6.4)	4 (5.1)	
Type of maintenance therapy used				
Cyc-MMF-Pred immunosuppressive therapy	7 (26.9)	15 (48.4)	25 (32.1)	0.016 (a)
Tac-MMF-Pred immunosuppressive therapy	6 (22.2)	9 (29)	17 (21.8)	
Tac-Aza-Pred immunosuppressive therapy	3 (11.5)	0	6 (7.7)	
Cyc-Pred immunosuppressive therapy	2 (7.7)	2 (6.5)	7 (9)	
Cyc-Aza-Pred immunosuppressive therapy	2 (7.7)	5 (16.1)	19 (24.4)	
Cyc-Eve-Pred immunosuppressive therapy	1 (3.8)	0	2 (2.6)	
MMF-Syr-Pred immunosuppressive therapy	5 (19.2)	0	1 (1.3)	
MMF-Eve-Pred immunosuppressive therapy	1 (3.8)	0	0	
Tac-Eve-Pred immunosuppressive therapy	0	0	1 (1.3)	

*number (%)

**The reported p value is based on the comparison of the three groups of diseases

DR= HLA-DR; Cyc= cyclosporine; Aza= azathioprine; MMF= mycophenolate; Tac= tacrolimus; Pred= prednisolone, Eve, everolimus, Syr= sirolimus; GFR= glomerular filtration rate; SD= Standard deviations

a. Pearson's Chi-squared test.

b. One-way ANOVA

c. Kruskal-Wallis test

### Survival

During the follow up, 18 patients (rate of 3.9%/person-year) died. The highest number of deaths was recorded in the DN-ESRD group with 13 patients (rate of 5.5%/person-year), nine due to a cardiovascular disease, three from an infection and one from a neoplasm. In the LN group, two patients (rate of 1.9%/person-year) died, both due to an infection. In the PCKD group, three patients (rate of 2.5%/person-year) died, one due to a cardiovascular complication and two from an infection; mortality with a functioning graft was observed in 11 patients (rate of 2.4%/person-year). 

**Figure 1.**
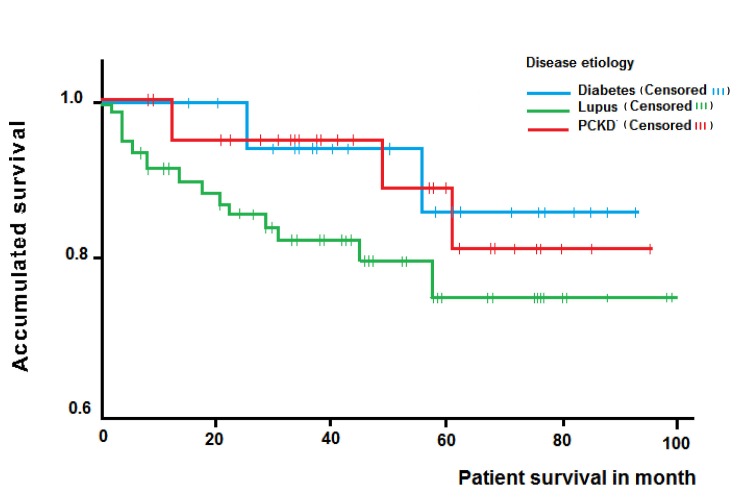
Kaplan-Meier curve for patient survival of renal transplant patients according ethiology of chronic kidney disease. (PCKD= polycystic kidney disease)

Overall survival rates of 94.4%, 88.5% and 79.6% were observed after one, three and five years, respectively. Subgroup analysis revealed patient survivals after one, three and five years of 100%, 87.5% and 87.5% for LN; 96.4%, 96.4% and 81.8% for PCKD and 91.7%, 82.4% and 75.7% for DN, respectively. The differences observed when analyzing the survival time according to the disease were not significant (log-rank test= 0.52) (Fig. 1).

On the final follow up, 26 graft losses (rate of 5.9%/ person-year) were reported, 18 from deceased patients, two from acute rejections, two due to infections, three from chronic graft nephropathy and one due to vascular complications.

**Figure 2.**
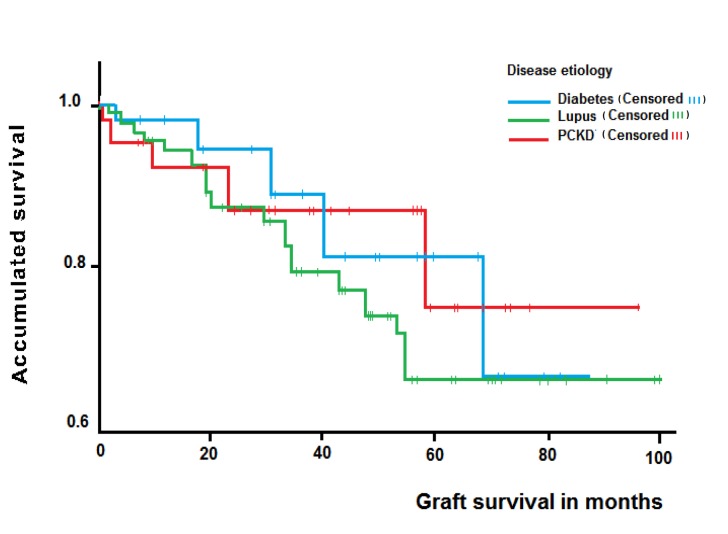
Kaplan-Meier curve for death censored graft survival of renal transplant patients according ethiology of chronic kidney disease. (PCKD= polycystic kidney disease).

Overall death-censored graft survival rates of 93.3%, 86.9% and 70.8% were observed after one, three and five years, respectively. The subgroup analysis exhibited graft survival rates of 96.3%, 82.5% and 82.5% for LN; 90%, 86.0% and 76.5% for PCKD and 91.7%, 80.3% and 67.9% for DN after one, three and five years, respectively, without a significant difference (log-rank test= 0.488) (Fig. 2).

An adjusted Cox regression model was performed with the significant variables in the univariate analysis and for the risk factors previously reported in the literature. Table 2 shows the variables associated with graft survival; only the black race (HR= 9.1; 95% CI= 2.67-31.1) and a previous graft rejection (hazard ratio: 3.97; 95% CI: 1.76-8.94) are associated with a lower graft survival rate. The etiology of ESRD was unrelated as a risk factor in decreasing the graft survival time (HR= 1.43; 95% CI= 0.52-3.93). 

**Table 2.** Association of the variables with the survival of the graft according to Cox regression.

Variables	HR	SE	p	95% CI
DR incompatibility equal to 1	1.28	1.06	0.814	0.16-10.29
DR incompatibility equal to 2	1.03	1.08	0.977	0.12-8.58
Cold ischemia time	1.01	0.04	0.821	0.93-1.09
Race	9.11	0.63	0.000	2.67-31.11
Rejection of the graft	3.97	0.41	0.001	1.76-8.94
Lupus vs non-lupus diagnosis	1.43	0.52	0.489	0.52-3.93

Log likelihood= 209.026. LR Chi-square= 31.4

HR= Hazard Ratio; SE= standar error; CI= Confidence interval; DR: HLA DR

### Evolution of the function of the kidney graft

**Figure 3.**
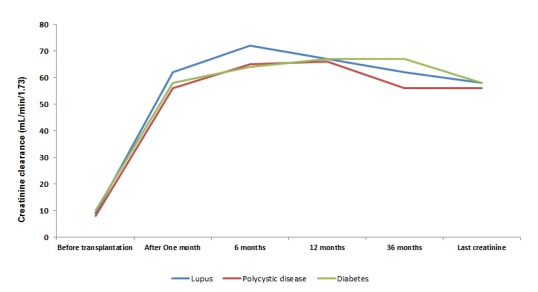
Glomerular filtration rate during follow up, according etiology of chronic kidney disease. Note that the times shown on the x axis no have a proportionality, the figure show trends of clearance creatinine only.

The glomerular filtration rate (GFR) using CKD-EPI was evaluated after one month, six months, one year, and three years as well as after a final registered follow-up, showing non-significant differences among the three groups (LN, PCKD, DN) (Fig. 3). 

The median GFR using CKD-EPI after three years of follow up was 62 mL/min/1.73 (SD: 19.4) in patients with LN, 56.4 mL/min/1.73 (SD: 20.8) in PCKD and 66.9 mL/min/1.73 (SD: 18.16) in DN (*p*= 0.17) (Fig. 3). Only one case of recurrence confirmed by biopsy was observed in the kidney transplant patients with LN (rate of 0.94%/person-year).

### Complications

Table 3 shows the main complications according to the etiology of ESRD. Infections, like urinary and cytomegalovirus infections, and delayed graft function were the most frequent complications. Some differences were observed when grouping by ESRD etiology, as more frequent risk of graft thrombosis in the PCKD group, more frequent risk of infection of the surgical wound, Cytomegalovirus (CMV) infections and delayed graft function in the DN group, and more frequent urinary infections in the LN group were observed. The DN group showed more frequent acute myocardial infarction.

**Table 3.** Rate of Complications according to the etiology of end stage renal disease

Variables*	Lupus (n= 27)	Polycystic disease (n= 31)	Diabetes (n= 78)
Delayed function of the kidney graft	1 (0.94)	4 (3.32)	10 (4.23)
Thrombosis of the graft	0 (0)	2 (1.66)	0 (0)
Fistula	1 (0.94)	3 (2.48)	2 (0.85)
Ureteral necrosis	1 (0.94)	1 (0.83)	1 (0.42)
Infection of the surgical wound	0	1 (0.83)	10 (4.23)
CMV infection	3 (2.82)	4 (3.32)	15 (6.35)
Urinary tract infections	19 (17.87)	17 (14.1)	33 (13.97)
Tuberculosis	1 (0.94)	1 (0.83)	3 (1.27)
Acute myocardial infarction	1 (0.94)	0 (0)	5 (2.12)
Cerebrovascular disease	0 (0)	1 (0.83)	3 (1.27)
Tumoral disease	0 (0)	1 (0.83)	1 (0.42)
Graft failure	5 (4.7)	5 (4.15)	16 (6.77)
Acute rejection confirmed by a biopsy	10 (9.4)	8 (6.63)	16 (6.77)
Mortality	2 (1.88)	3 (2.48)	13 (5.5)

* Rates: % person-year

CMV: cytomegalovirus

Transplant rejection was confirmed by biopsy in 34 (rate of 7.3%/person-year) patients with no significant differences among the etiological groups of ESRD in the number of rejections (*p*= 0.23). Graft failure was observed in five patients with an LN diagnosis (rate of 4.7% person-year) (Two due to death and three from chronic transplant nephropathy); 16 (rate of 6. 8%/person-year) in the DN group (13 due to death, two from an infection and one acute rejection); and five (rate of 4.2%/person-year) in the PCKD group (one due to acute rejection, one due to a vascular cause and three due to death). 

## Discussion

This study was performed at a single kidney transplant center in the city of Medellin-Colombia and aimed to evaluate the patient and graft survival rates in a kidney transplant population with LN-ESRD and to compare these rates with PCKD- and DN-kidney transplant, patients with a best and worse prognosis after kidney transplantation respectively. As our main finding, significant differences were not observed in the patient or graft survival rates among the groups (DN, LN and PCKD).

Patients with DN-ESRD were considered the comparison group due to the association of their comorbidities with a worse prognosis, a lower graft survival, a greater incidence of cardiovascular events and early mortality in contrast to other kidney transplant populations 19,20,22,24. The second comparison group was PCKD-ESRD patients due to the association with minor systemic compromise and comorbidities with fewer complications and similar graft and patient survival rates compared to other control groups in different studies 25-30

Our graft and patient survival rates were similar to those in other studies, showing comparable rates in LN-ESRD patients and non-lupus patients 14,31. Mojcik *et al., * reported a 87.4% and 63.1% patient and graft survival for LN patients, respectively 4. Bumgardner *et a.,l* showed similar results in LN-ESRD kidney transplant patients and ESRD due to other causes 14. Ponticelli *et al.*, in a study on 32 LN kidney transplant patients, reported a 96% patient survival after ten years vs 91% in controls and 77% graft survival in LN vs 78% in controls 11. Fuentes *et al* performed a study on 94 LN kidney transplant patients and a group of DN-ESRD, showing a similar curve of graft survival in five years for both groups 32. Nonetheless, other studies show worse results in the graft survival of LN patients 33,34. Using the data from the United States Renal Data System (USRDS), Ward *et al* showed an increase in the risk of graft failure in LN patients after adjusting for confounders (HR= 1.08; 95% CI= 0.94-1.23; *p*= 0.28) 35. Stone *et al* reported a graft survival of 81.7%, 74.7%, 45.9% and 18.5% in a LN kidney transplant group vs 88.2%, 84.4%, 45.9 and 34.8% ESRD kidney transplant patients secondary to other causes after one, two, five and ten years, respectively; in this last study, kidney failure was reported in 49.1% of the LN patients and in 34.9% of the controls 33,36,37.

Differences were not significant in the outcome of the PCKD group compared with those in the other two groups. Nonetheless, Jhonston *et al*, in 2005, reported kidney graft survival rates in PCKD patients of 88.2%, 79.1% and 54.3% vs 85.5%, 68.1% and 48.6% in controls after one, five and ten years, respectively. These differences were significant 38. Jacquet *et al* reported similar patient survival rates in a study on 534 PCKD-KT patients and 4,779 non-PCKD KT patients as follows: 93.4% vs 93.4% after five years, 87.4% vs 87.2% after ten years and 78.7% vs 82.4% after 15 years, respectively (*p*= 0.464). However, the death-censored graft survival rates were higher in the PCKD group (90.4% vs 86.9% after five years; 81.1% vs 75.4% after ten years and 76% vs 66% after 15 years *p*= 0.0187) 26.

In our study, patient survival was lower in the DN population than in the LN and PCKD groups (a 75.5% survival after three years in the DN group vs 87.5% in the LN group and 81.8% in the PCKD group); however, this difference was not statistically significant possibly due to the small sample size of the LN and PCKD groups. Older patients in the diabetic group and significantly more cardiovascular comorbidities (coronary disease and chronic occlusive arterial disease) would explain this tendency of an increase in the risk of cardiovascular complications and mortality. Previous studies have also reported this greater risk of cardiovascular diseases and mortality in diabetic patients 24. Rocha *et al*., compared 62 diabetic kidney transplant patients vs 62 non-diabetic patients over an average follow-up period of 102 months, observing patient survival rates of 60% and 50% after five and ten years for diabetics and 96% and 84% for non-diabetics, respectively (*p* <0.001) 24. Cosio *et al*., evaluated the impact of DM in the morbidity and mortality of kidney transplanted patients; in their results they found a decreased survival and increased incidence of cardiovascular events, being these the principal cause of patient death in this population 61 vs 26% in non diabetic population; an increased mortality associated to infection was found as well 23,39. 

Previously, kidney transplantation in LN-ESRD patients was considered a contraindication due to a high probability of recurrence of the disease and higher morbidity. This notion was reconsidered approximately 30 years ago after some reports demonstrated that the KT survival rate was similar in patients with LN-ESRD and transplant patients from other causes. Thereafter, KT has been accepted as a treatment option for LN-ESRD patients 40-43. To date, the long-term prognosis and recurrence rate of nephritis continues to be controversial because different studies have reported recurrence between 0 and 30% 10,12,15,43-45. In our study, the rate of recurrence of LN was very low (0.98%/person-year); however, in our group we do not perform protocol biopsies after kidney transplantation and that might cause under-reporting of this datum 4,11. Yu *et al.,* observed a recurrence of 26.1% in 32 LN kidney transplant patients, with 50% considered as class 1, and no negative impact on graft survival 12.

The variables reported previously as risk factors for graft survival were included in our series. When performing a Cox regression model, a history of acute rejection and black race were the only risk factors associated with a decrease in graft survival. In turn, age, the number of HLA incompatibilities, time in dialysis, time in cold ischemia, type of immunosuppression therapy and the disease causing ESRD (LN, PCKD and DN) did not affect graft survival. Previous studies have reported that transplant rejection is another important risk factor in graft failure because it activates T cells, leading to an inflammatory response that will eventually deteriorate the function of the kidney graft 15.

Regarding race, previous studies have considered the black race as a risk factor in the progression of kidney disease and for a reduced response to immunosuppressive therapy 45; hence, African-Americans have an increased risk of rejection 6. Nonetheless, previous studies, including the study by Contreras *et al.*, comparing African-American and Caucasian LN KT patients showed that race was not a predictive factor of graft failure when other variables are considered 45. Nee *et al.,* evaluated graft survival in 4,214 LN kidney transplant patients comparing African-Americans vs other races. These authors found an increased risk of graft failure and death in African-Americans even after adjusting for variables such as income 6. In our study, the black race was a risk factor for reduced graft survival; however, conclusions are not definite because the black population represented a small portion of the sample.

Furthermore, several studies have considered that the type of donor (living vs deceased) affects graft survival. Previous studies have indicated better results in living-donor transplants (69.1%) vs deceased-donor transplants (56.3%) 4. Data from the USRDS demonstrate, in lupus patients, a five-year graft and patient survival of 77% and 94%, respectively with living-donor transplants vs 58.1% and 83.8% with deceased-donor transplants, respectively 46. These data could not be confirmed in the present study because our institution performs most kidney transplants from deceased donors. 

When evaluating the main complications and grouping them according to the etiology of CKD, significant differences were not found. Previous studies have suggested a higher risk of vascular disease, increasing the morbimortality in KT patients secondary to LN and antiphospholipid syndrome 11. Furthermore, this group had a higher risk for neoplasias, such as non-Hodgkin Lymphoma, and skin and bladder squamous cell carcinoma, which are often associated with the previous use of cyclophosphamide 47,48. These findings were not obtained in the present study, although they may be due to the small sample. Nonetheless, although the sample size may have affected our results, a slight significant difference was observed with graft thrombosis, which was higher in PCKD patients, however was no found an answer for this; infections on the surgical wound which was higher in diabetics, it is explained because diabetic group has more risk for infection diseases; and urinary infections which was higher in lupus patients because it often presented in women, and feminine gender is a risk factor for urinary tract infection.

The limitations of this study include it being conducted at only one center, the limited sample size and its retrospective nature. The size of the sample may have influenced the assessment of the effect of the variables and impact on survival. However, although the analysis was adjusted for the comorbidities, not all variables involved in the results could be identified or measured, besides the heterogeneous population with different immunosuppression regimens, it made very difficult to assess the impact of this variability on the graft outcome; thus, conclusions may have a limited statistical power. However, there are not previously series comparing these three groups. In order to know the true prognosis in LN KT patients, it was compared it with DN KT because they have bad prognosis, and also we compared with PCKD KT patients because they have better prognosis.

## Conclusion

Although the small sample size limits the ability to make definitive interpretations of these data, the results of this study suggest that LN-ESRD patients undergoing KTs have a similar graft and patient survival success rate to that in other groups such as patients with DN and PCKD. The complication rate and risk of recurrence of the disease were low, and the glomerular filtration rate was similar in the three groups of the study. All this suggests that Kidney transplants should be considered as the treatment of choice for patients with ESRD secondary to LN.
